# Multivitamin, mineral and n-3 pufa supplementation to reduce aggression among chronically admitted psychiatric patients: A randomized clinical trial

**DOI:** 10.1192/j.eurpsy.2021.444

**Published:** 2021-08-13

**Authors:** N. De Bles, N. Rius Ottenheim, J. Geleijnse, O. Van De Rest, J. Bogers, W. Van Den Hout, A. Van Hemert, E. Giltay

**Affiliations:** 1 Psychiatry, LUMC, Leiden, Netherlands; 2 Division Of Human Nutrition And Health, Wageningen University & Research, Wageningen, Netherlands; 3 High Care Clinics, MHO Rivierduinen, Leiden, Netherlands; 4 Biomedical Data Science, LUMC, Leiden, Netherlands

**Keywords:** n-3 PUFA, Aggression, psychiatric inpatients, nutritional supplements

## Abstract

**Introduction:**

Aggression and violent incidents are a major concern in psychiatric inpatient care, potentially leading to physical and psychological consequences for both patients and staff. Nutritional supplementation was found to reduce aggressive incidents and rule violations in forensic populations and children with behavioural problems.

**Objectives:**

To assess whether multivitamin, mineral and n-3 PUFA supplementation is effective in reducing the number of aggressive incidents among psychiatric patients who are chronically admitted.

**Methods:**

In a pragmatic, multicentre, randomized, double-blind, placebo-controlled study, psychiatric inpatients were randomized to receive either three supplements containing multivitamins, minerals, and n-3 PUFA or placebo. During the intervention period of six months, aggressive incidents were assessed using the Staff Observation Aggression Scale – Revised (SOAS-R). Secondary outcome parameters were the patients’ quality of life and affective symptoms. The trial was registered in the Clinical Trials Register (NCT02498106).

**Results:**

A total of 176 patients were enrolled and randomly assigned to receive supplements (n=87) or placebo (n=89). They were on average 49.3 years old (SD=14.5), and 64.2% were male. Most patients had a psychotic disorder (60.8%). Supplementation versus placebo significantly increased circulating micronutrient levels. The primary outcome of SOAS-R incidents was similar in those assigned to supplements (1.03 incidents per month; 95% confidence interval [CI]: 0.74-1.37) and placebo (0.90; 95%CI: 0.65-1.19), with a rate ratio of 1.08 (95%CI: 0.67-1.74; p=0.75). Differential effects were neither found in sensitivity analyses on the SOAS-R, nor on secondary outcomes.

**Conclusions:**

Six months of nutritional supplementation did not reduce aggressive incidents among chronically admitted psychiatric inpatients.

**Disclosure:**

No significant relationships.

